# Phenotypic and genotypic characteristics of *Escherichia coli* strains isolated during a longitudinal follow-up study of chronic urinary tract infections

**DOI:** 10.3389/fpubh.2023.1240392

**Published:** 2023-11-22

**Authors:** Ulises Hernández-Chiñas, Ricardo E. Ahumada-Cota, Armando Navarro-Ocaña, María E. Chávez-Berrocal, José Molina-López, Luz M. Rocha-Ramírez, Armando Navarro-Cid del Prado, Carlos A. Eslava

**Affiliations:** ^1^Research Division, Public Health Department, Peripheral Unit of Basic and Clinical Research in Infectious Diseases, Faculty of Medicine, UNAM, Mexico City, Mexico; ^2^Bacterial Pathogenicity Laboratory, Hemato-Oncology and Research Unit, Children’s Hospital of Mexico Federico Gómez, Mexico City, Mexico; ^3^Bacteriology Laboratory, Public Health Department, Faculty of Medicine, UNAM, Mexico City, Mexico; ^4^Unidad de Investigación en Enfermedades Infecciosas, Hospital Infantil de México Federico Gómez, Secretaría de Salud, Mexico City, Mexico; ^5^Faculty of Chemistry, National Autonomous University of Mexico, Mexico City, Mexico

**Keywords:** uropathogenic *Escherichia coli*, persistent UTI, reinfection UTI, urovirulence genes, drug-resistance

## Abstract

Worldwide, Urinary Tract Infections (UTIs) are an important health problem with many cases reported annually, women being the most affected. UTIs are relevant because they can become a recurrent condition, associated with different factors that contribute to the chronicity of the disease (cUTI). cUTI can be classified as persistent (peUTI) when the causative agent is the same each time the infection occurs or as reinfection (reUTI) when the associated microorganism is different. The purpose of this work was to characterize *Escherichia coli* isolates obtained in two prospective studies of patients with cUTI, to define which of them corresponded to peUTI and which to reUTI. A total of 394 isolates of *E. coli* were analyzed by agglutination with specific sera, antimicrobial susceptibility by diffusion disc test, and the phylogroups and presence of genes associated with virulence by PCR assays. Additionally, in some characterized strains adherence, invasiveness, and biofilm formation were analyzed by *in vitro* assays. The results showed that the peUTI strains belonged mainly to the classical UPEC serogroups (O25, O75, O6), were included in the B2 phylogroup, carried a great number of virulence genes, and were adherent, invasive, and biofilm-forming. Meanwhile, reUTI strains showed great diversity of serogroups, belonged mainly in the A phylogroup, and carried fewer virulence genes. Both peUTI and reUTI strains showed extensively drug-resistant (XDR) and multidrug-resistant (MDR) profiles in the antimicrobial susceptibility test. In conclusion, it appears that peUTIs are caused principally by classical UPEC strains, while reUTIs are caused by strains that appear to be a part of the common *E. coli* intestinal biota. Moreover, although both peUTI and reUTI strains presented different serotypes and phylogroups, their antimicrobial resistance profile (XDR and MDR) was similar, confirming the importance of regulating prophylactic treatments and seeking alternatives for the treatment and control of cUTI. Finally, it was possible to establish the features of the *E. coli* strains responsible for peUTI and reUTI which could be helpful to develop a fast diagnostic methodology.

## 1 Introduction

With the development of antibiotic therapy in the mid-20th century, the clinical impact and devastating effect of infectious diseases was controlled. However, the inappropriate use of antimicrobials in the treatment of these diseases and their use in areas such as agribusiness and others, has been the cause of a significant increase of antimicrobial resistant bacteria, causing global population to currently face a major public health problem (1). Urinary Tract Infections (UTIs) represent a major health problem worldwide (2), predominantly more common in women and, although, UTIs occur at any stage of life, their highest incidence occurs between the ages of 15 and 24 years-old and in the post-menopausal period (3). The higher frequency of UTI in women has been linked to the proximity between the anus and the urethra, a situation that facilitates the contamination of the latter by feces (2, 4). Another relevant aspect of UTIs is their high rate of recurrence, thus becoming a chronic condition and affecting the quality of life due to the condition itself and the emotional and psychological effect it entails. In addition to the negative effects, their frequency with which cUTIs occur has an impact on the patient’s economy, either because of the medical costs or the need to be absent from work (5, 6). cUTIs are characterized by recurrent infections, which in turn are defined as persistent infections (peUTI), when the responsible microorganism is always the same; or reinfection (reUTI), in cases where the microorganism is different each time a new UTI occurs. To explain the etiology of both models it is pointed out that in peUTI, after the initial infection the pathogen establishes itself and resides within the epithelium of the urinary tract, avoiding the immune response of the host and protecting itself from the effect of antimicrobials (7). While the diversity of bacteria related to reUTI may be explained by the lack of genetic information that allows bacteria to maintain a stable form within the epithelium of the urinary tract, and therefore they can be eliminated more easily. However, depending on the patient’s susceptibility, they can be infected again through fecal contamination of the urethra by common intestinal microorganisms, or by some other mechanisms related to the patient’s poor hygienic habits (4). The treatment for both peUTI and reUTI is the prescription of antimicrobials, whose constant use alters the normal microbiota of the bladder, vagina, and gastrointestinal tract, favoring the selection and proliferation of multidrug-resistant strains. *Escherichia coli* is the main bacterial agent associated with UTI and is responsible for 70–95% of community-acquired infections and approximately 50% of nosocomial UTI cases (8–10). *E. coli* is a bacterium with a genome that presents great plasticity, a fact that has favored its great diversity formed by commensal clones that naturally colonize the intestine and contribute to the proper functioning of the host organism. However, in the evolutionary process, *E. coli* has generated pathogenic clones (pathotypes) that can cause intestinal diseases called diarrheogenic (DEC), and others more related to infections outside the intestine, the case of UTIs, named extraintestinal *E. coli* (ExTEC). The phenotypic diversity of *E. coli* is related to the composition of the different somatic (O) and flagellar (H) antigens expressed by the bacterium; both react to specific sera allowing to classify strains in different serogroups (O antigen only) and serotypes (both O and H antigens). By this method, some *E. coli* strains associated with UTIs have been designated as classic uropathogenic *E. coli* (UPEC) (11, 12). In the laboratory, we have the complete sera scheme to perform the antigenic characterization of *E. coli*, which allows us to know the serogroup and serotype of the bacterium and thus define the variety that causes the different types of UTIs. Previously, in two prospective studies conducted by our research group, urine samples from patients with cUTI were collected and analyzed monthly, followed up for a period of seven to 18 months, varying depending on the patient. From each positive urine culture, 10 colonies were selected, identified using biochemical tests, and serotyped; the strains were frozen for preservation and subsequent characterization (13, 14). The aim of this study was to perform the phenotypic and genotypic characterization of some of the *E. coli* strains previously isolated from the already mentioned studies, with the interest of analyzing the behavior of the isolates obtained from patients with peUTI and reUTI and thus propose strategies for the diagnosis, treatment, and potential control of cUTI.

## 2 Materials and methods

### 2.1 Bacteria

The study included 394 *Escherichia coli* isolates obtained from 131 urine samples from 39 adults and 17 children who participated in the two previously mentioned prospective studies of cUTI (13, 14).

### 2.2 Serotyping of *Escherichia coli* isolates

*Escherichia coli* strains were serotyped in a previous work (13, 14). In brief, the agglutination assays were performed using 96-well microtiter plates and rabbit antisera against O1 to O187 somatic (O) antigens and 53 flagellar (H) antigens prepared in rabbits (SERUNAM, registered trademark in Mexico, number 323,158/2015) using the method described by Orskov and Orskov (15).

### 2.3 Antimicrobial susceptibility

To assess antimicrobial susceptibility, the disk diffusion method was performed following the protocol described by Clinical and Laboratory Standards Institute (CLSI) (16). Susceptibility to 34 antimicrobials (Oxoid, United Kingdom) was assessed for 125 and 269 isolates from peUTI and reUTI, respectively. Discs impregned with ampicillin (AMP) 10 μg, piperacillin (PRL) 100 μg, carbenicillin (CAR) 100 μg, mecilanam (MEL) 10 μg, amox-clavulanic acid (AMC) 20–10 μg, piperacillin-tazobactam (TZP) 100–10 μg, cefazolin (KZ) 30 μg, cephalothin (KF) 30 μg, cefamandol (MA) 30 μg, cefepime (FEP) 30 μg, cefoperazone (CFP) 75 μg, cefoxitin (FOX) 30 μg, ceftriaxone (CRO) 30 μg, ceftazidime (CAZ) 30 μg, furoxime (CXM) 30 μg, meropenem (MEM) 10 μg, nitrofurantoin (F) 300 μg, aztreonam (ATM) 30 μg, gentamicin (CN) 10 μg, amikacin (KA) 30 μg, kanamycin (K)30 μg, tobramycin (TOB) 10 μg, streptomycin (S) 10 μg, tetracycline (TE) 30 μg, ciprofloxacin (CIP) 5 μg, norfloxacin (NOR) 10 μg, trimethoprim-sulfamethoxazole (SXT) 1.5/23.75 μg, nalidixic acid (NA) 30 μg, sulfonamides (S3) 250/300 μg, trimetroprim (W) 5 μg, chloramphenicol (C) 30 μg, fosfomycin (FOS) 200 μg, fosfomycin trometramol (FOT) 200 μg were used. The results were interpreted considering the diameter of the inhibition halo, categorized as susceptible (S), intermediate (I) and resistant (R) according to the CLSI (16) criteria, the *E. coli* ATCC 25922 reference strain was used as a control. Isolates classified as intermediate were re-classified as resistant, multidrug-resistant (MDR) strains were defined as: resistant to ≥1 agent in ≥3 antibiotic categories, extensively drug-resistant (XDR): resistant to ≥1 agent in all but <2 categories and pandrug-resistant (PDR) when resistant to all antimicrobial agents used (17). For each isolate, a resistance score was calculated, defined as the number of antibiotics to which that strain was resistant among the 34 antibiotics tested (18).

### 2.4 PCR assays

#### 2.4.1 DNA extraction

DNA extraction was performed according to the manufacturing instructions of InstaGene Matrix kit (Bio-Rad, United States).

#### 2.4.2 Phylogenetic analysis

Using the multiplex PCR method described by Clermont et al. (19), primers for *chuA*, *yejA*, and TspE4.C2 were used to identify the phylogenetic group of the different *E. coli* isolates (A, B1, B2, and D).

#### 2.4.3 Virulence genes

Multiplex PCR (endpoint) was used to analyze the presence of 15 virulence genes associated with adhesion proteins (*fimH*, *papA*, *papC*), toxin production (*sat*), iron uptake (*feoB*, *ireA*, *irp-2*, *sitA*, *iutA*, *fyuA*), capsule synthesis (*kpsMT-K1*), pathogenicity island I (*malX*), and the enzyme associated with the degradation of antimicrobial peptides (*ompT*). Simplex assays were performed for sat, *papA*, *ompT*, *iroND*, *malX*, and duplex assays for the combinations (1) *ibeA*, *iutA*; (2) *feoB*, *sitA*; (3) *fimH*, *ireA*; (4) *irp-2*, *kpsMTII* and (5) *fluA* and *fluB*. The sequences of the primers are listed in Supplementary Table S1, PCR assays were performed under the following conditions: final volume 10 μL, reaction mixture consisting of 1.0 μL DNA, 0.4 μL (10 μM) of each primer, 5 μL (2X) PCR Master Mix (Thermo Scientific. United States). Amplification was performed using a MiniAmp thermal cyclerTM (Applied Biosystems., United States) according to the following conditions: denaturation step for 2 min (95°C); amplification of 30 cycles for 30 s (95°C), annealing temperature for 30 s, 1 min at 72°C and a final extension step for 7 min at 72°C (Supplementary Table S1). PCR products were analyzed by electrophoretic run using agarose gels (1.2%), stained with ethidium bromide (0.01%), and were visualized using a Cleaver Scientific TTD model OmniDoc Gel Documentation System (United Kingdom) ultraviolet light transilluminator. A virulence score was calculated for each isolate, defined as the number of virulence genes present in the strain with respect to the 15 genes analyzed (18).

### 2.5 *In vitro* assays

Some of the studied *E. coli* strains were selected to evaluate their *in vitro* properties in terms of adherence, invasiveness, and biofilm formation capacity to subsequently identify if there was a correlation between these properties and the cUTI type.

#### 2.5.1 Adherence to cells

For this assay, 72 strains isolated from 37 urine cultures belonging to 12 patients with reUTI and 7 with peUTI who presented a positive urine culture for >3 months were selected. The presence of genes associated with fimbrial adhesins was the selection criterion for the *E. coli* strains used in this assay. The procedure described by Cravioto et al. (20) was used with some modifications. Briefly, 1 mL (2.5×10^5^) of HEp-2 cells in suspension (ATCC CCL-23) was plated in a 24-well tissue culture microplate (Costar® United States) containing sterile 13 mm slides (Nunc Brand Products® United States) and Minimum Essential Medium (MEM) (Invitrogen. United States), supplemented with 10% Fetal Bovine Serum (FBS) (EquiLab, Canada). Cells were incubated for 24 h at 37° C in a 3% CO_2_ atmosphere, upon reaching 90% confluence the medium was removed and washed three times with sterile Phosphate-Buffered Saline (PBS) (1x), the bacterial inoculum was prepared from a previous culture (grown overnight) by adjusting the suspension to a concentration of 3.0 × 10^8^ CFU/mL in MEM without FBS and antibiotic, and supplemented with 100 μL of 10% D-mannose, 1 mL of this was added to each well. The plates were incubated for 3 h at 37°C in 3% CO_2_ atmosphere, at the end of this time the medium with bacteria was removed and wells were washed twice with 1x PBS, the cell monolayer was fixed with methanol for 10 min and finally stained with 1% Giemsa. The number of adherent bacteria was counted independently by two persons using a microscope (100X), analyzing at least 15 fields of each preparation. The result was expressed as the average number of adherent bacteria per cell per duplicate assay. The following strains were used to evaluate adherence phenotypes: *E. coli* E2348/69 (localized adherence), *E. coli* 87,125 (diffuse adherence), *E. coli* 49,766 (aggregative adherence) and a non-adherent *E. coli* strain HB101.

#### 2.5.2 Cell invasion

In this assay, 54 strains isolated from 27 urine cultures from 7 peUTI and 10 reUTI patients who had a positive urine culture for more than 3 consecutive months were tested. *Shigella boydii* 21639 and *E. coli* 1124 were used as positive controls and *E. coli* HB101 as a negative control. For invasion assays, the procedure described by Elsinghorst (21) with modifications was used. Briefly, HEp-2 cells (ATCC CCL-23) were cultured and infected following the same conditions described in the adherence assay. In this assay a first incubation of 3 h was performed, the medium was removed from the wells and washed three times with sterile PBS to eliminate the bacteria that failed to invade, 1 mL of MEM with Gentamicin (100 mg/mL) and lysozyme (300 mg/mL) was added, the plates were incubated again for 3 h at 37°C (22). At the end of the new incubation period the culture was washed twice with 1x PBS and the culture was fixed with methanol for 15 min and stained with 1% Giemsa for 20 min. The preparations were observed under the microscope and the cells with intracellular bacteria were counted. A bacterial quantification test was performed, briefly the culture medium was removed and 500 μL of Triton (0.1%) was added to each well for 15 min at room temperature to break the HEp-2 cells. The suspension obtained was subjected to serial ten-fold dilutions and plate count was performed using the drop-plate technique (10 μL) on MacConckey agar. The assay was repeated in two independent experiments, and the data analyzed was the average of invasive bacteria.

#### 2.5.3 Biofilms

The biofilm formation capacity of 80 UPEC strains, 68 selected from 10 patients with peUTI and 12 from 3 patients with reUTI, all with a follow-up of more than 3 months, and with the presence of adherence-related genes, was analyzed. The method described by Christensen et al. (23) was used with minor modifications. Briefly, bacteria were inoculated in Luria Bertani broth (LB) and incubated at 37°C with constant shaking (200 rpm) until a 1 McFarland turbidity standard suspension (corresponding to 3×10^8^ CFU/mL) of each isolate was reached. 50 μL volume of the previous suspension was placed in duplicate in 24-well plates (COSTAR, United States), 950 μL of MEM was added to each well and incubated at 37°C for 24, 48 and 72 h. The *E. coli* CFT073 strain was used as a positive control and *E. coli* HB101 as a negative control; as a blank, a well was used only with MEM without bacteria; the process was carried out under the same conditions used for the wells with bacteria. After each incubation time, the medium was removed from each well, washed twice with sterile water and dried at room temperature for at least 30 min. To each well, one milliliter of crystal violet solution (1%) was added and incubated for 20 min at room temperature. The dye was removed, and the wells were washed twice with distilled water. Once dry, one milliliter of 96% ethanol was added to each well and mixed using a micropipette, biofilm formation was defined by measuring O.D. at 570 nm using a microplate reader (Spectronic GenesysTM 2). All measurements were performed in two independent experiments, biofilm formation was classified as strong biofilm formers (4DOc < DO), moderate (2DOc < DO≤4DOc), weak (DOc<DO≤DO≤2DOc), or non-biofilm formers (DO≤DOc) (24). Strains that showed biofilm production were analyzed to identify the presence of the *fluA* (primer F: 5′-aggcaggaggaactgccagt-3′ and R: 5′-taaatgagggtgggtgcccgtgcc-3′) and *fluB* (primer F: 5′-cagccggatctgcc-3′ and R: 5′-actctggtgtttctggctgtt-3′) genes, alleles of the Ag43 antigen, following the protocol described by Zalewska-Piatek et al. (25) and Danese et al. (26).

### 2.6 Statistical analysis

Statistical analysis was performed using GraphPad Prism version 8 software (GraphPad Sotfware, San Diego, CA, United States). The prevalence of virulence genes and antibiotic resistance patterns were compared between reUTI and peUTI by Fisher’s exact test. Virulence and resistance scores between groups (classical vs. Non-classical UPEC; Phylogroups; reUTI vs. peUTI) were compared by performing Student’s *t*-test. Additionally, linear regression was performed and Pearson’s correlation between virulence and resistance scores shown by the strains was calculated (18). For all analyses, a value of *p* < 0.05 was considered statistically significant.

## 3 Results

### 3.1 Serogroups and serotypes of the analyzed *Escherichia coli* strains

The serology of the 394 strains corresponded to 44 serogroups and 76 serotypes, 185 (47%) of the strains were included in 10 of the classical UPEC serogroups, 134 (34%) belonged to 34 different non-UPEC serogroups, 48 (12%) presented rough phenotype (R), and 28 (7%) of the strains did not agglutinate (ND, non-determined) with any of the 187 sera used (Table 1). The correlation between the type of UTI and serogroups, it was found that strains with classical UPEC serogroups belonged to patients with peUTI (*p* < 0.05). On the other hand, patients with reUTI, the strains belonged preferentially to non-UPEC serogroups or were not identified (*p* < 0.05). A more specific analysis showed that 52.5% of the strains belonged to the classical UPEC serogroups O25, O75, O8, O6, O1, and non-UPEC O9, O11, and O14 (Table 1). The serotype analysis in peUTI patients with a follow-up >7 months showed that O25: H4, O75: NM, O6: H1, and O9: NM were consistently isolated (*p* < 0.05). With respect to patients with reUTI the serotypes O8: NM, O8: H9, O1: H7, and O25: H4 were the most frequently identified. It was interesting to note that strains of serotype O25: H4 were isolated from both types of infections, peUTI and reUTI.

**Table 1:** *Escherichia coli* serogroups in patients with peUTI and reUTI.

	Serogroup (No. of strains)		
	reUTI, *n* = 269	peUTI, *n* = 125	Total, *n* = 394
Classical UPEC	**O25**^(21)^,**O8**^(15)^,**O1**^(10)^,**O6**^(8)^,**O16**^(6)^,**O4**^(3)^,**O21**^(3)^,**O22**^(3)^,**O75**^(3)^	**O25**^(69)^,**O75**^(36)^,**O6**^(6)^	**184** (47%)
Non-Classical UPEC	**O11**^(14)^,**O14**^(12)^,**O28ab**^(6)^,**O32**^(6)^,**O73**^(6)^,**O174**^(6)^,**O57**^(5)^,**O76**^(4)^,**O178**^(4)^,**O12**^(**3**)^,**O19**^(3)^,**O45**^(3)^,**O84**^(3)^,**O96**^(**3**)^,**O101**^(3)^,**O109**^(3)^,**O120**^(3)^,**O147**^(3)^,**O148**^(3)^,**O153**^(**3**)^,**O154**^(3)^,**O170**^(3)^,**boy8**^(3)^,**O17**^(2)^,**O23**^(2)^,**O152**^(2)^,**O49766**^(2)^,**O18ac**^(1)^,**O20**^(1)^,**O49**^(1)^,**O102**^(1)^,**O168**^(1)^	**O9** ^(11)^	**134** (34%)
Non-defined	**OR**^(45)^,**OND**^(28)^	**OR** ^(3)^	**76** (19%)

### 3.2 Antimicrobial susceptibility

**Figure 1:**
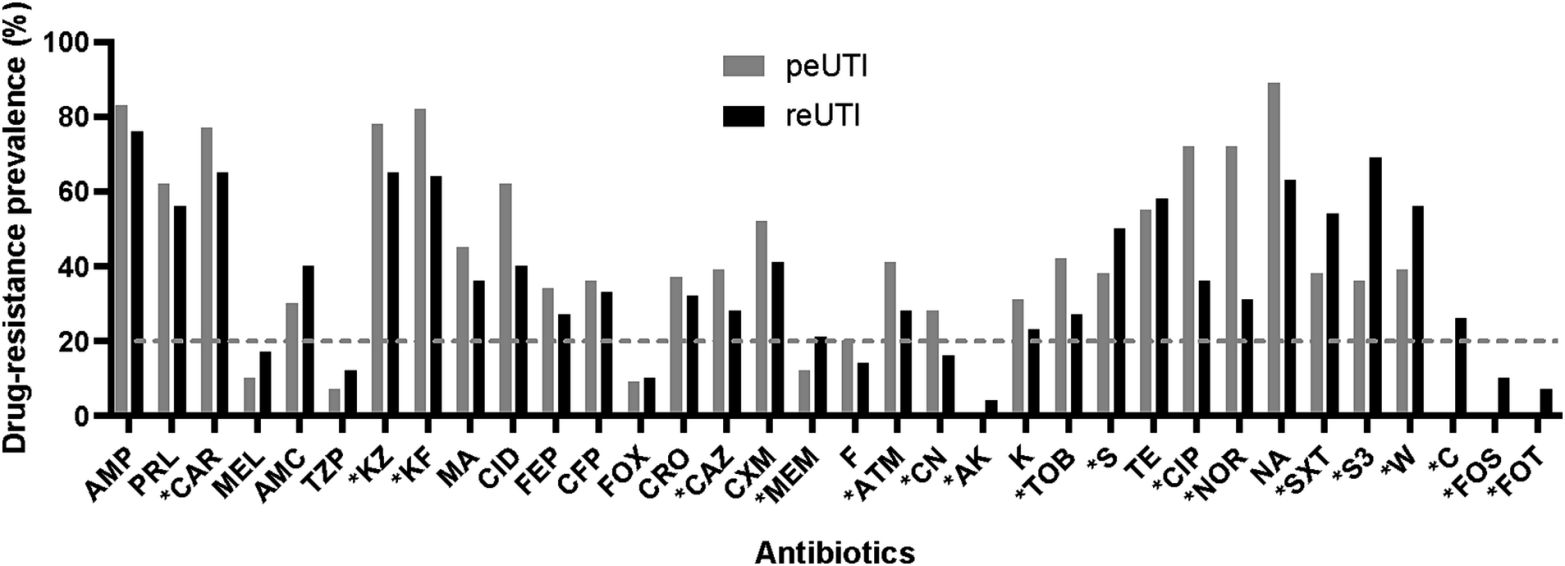
Drug-resistance prevalence among UPEC isolates from patients with reUTI and peUTI. Ampicillin (AMP), piperacillin (PRL), carbenicillin (CAR), mecilanam (MEL), amox-clavulanic acid (AMC), piperacillin-tazobactam (TZP), cefazolin (KZ), cephalothin (KF), cefamandol (MA), cefepime (FEP), cefoperazone (CFP), cefoxitin (FOX), ceftriaxone (CRO), ceftazidime (CAZ), furoxime (CXM), meropenem (MEM), nitrofurantoin (F), aztreonam (ATM), gentamicin (CN), amikacin (KA), kanamycin (K), tobramycin (TOB), streptomycin (S), tetracycline (TE), ciprofloxacin (CIP), norfloxacin (NOR), trimethoprim-sulfamethoxazole (SXT), nalidixic acid (NA), sulfonamides (S3), trimetroprim (W), chloramphenicol (C), fosfomycin (FOS), fosfomycin trometramol (FOT). The dotted line marks the 20% resistance threshold. Fisher’s exact test was performed, *p < 0.05.

**Figure 2:**
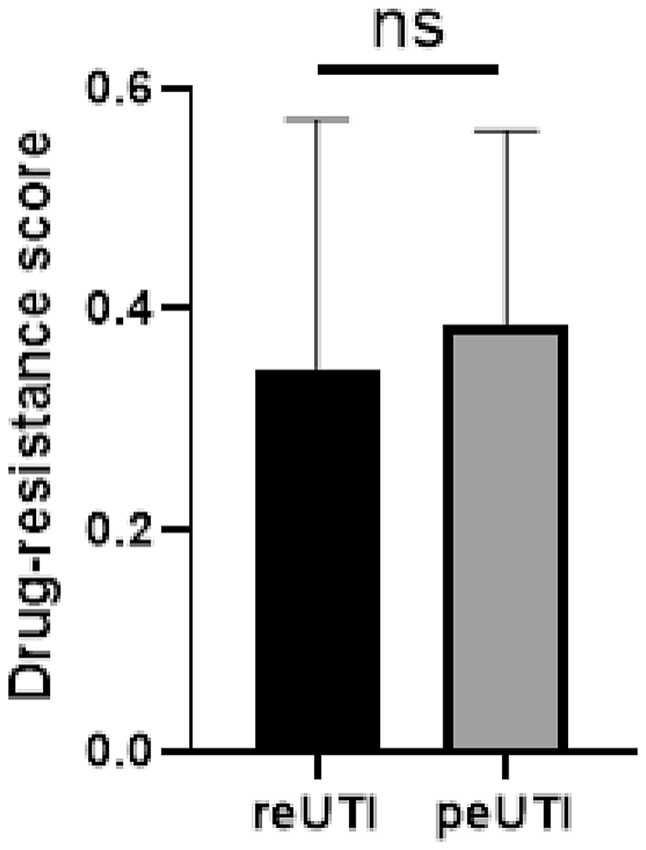
Distribution of the drug-resistance score in UPEC isolates from patients with reUTI and peUTI. The bars within each box plot show median values. The box covers the 25th percentile to the 75th percentile of the data. Bars above and below the box show 1.5 times the inter-quartile range. Whiskers show minimum and maximum values. Student’s *t*-test was performed, ns, statistically non-significant and *p < 0.05.

A relevant aspect in UTI is the poor response to antimicrobial treatment in a significant number of patients. The sensitivity results showed that 378 (96%) strains showed low resistance to mecilinam, piperacillin/tazobactam, cefoxitin, meropenem, amikacin, nitrofurantoin, chloramphenicol, fosfomycin, fosfomycin/trometramol. However, a different profile with high resistance to the penicillin family (ampicillin, piperacillin, carbenicillin), some cephalosporins (cefazolin, cephalotin) and nalidixic acid was identified in most strains (Figure 1). Comparative analysis showed significant higher resistance (*p* < 0.05) for carbenicillin, cefazolin, cephalotin, ceftazidime, aztreonam, gentamicin, tobramycin, ciprofloxacin, and norflozacin in peUTI strains and for meropenem, amikacin, streptomycin, trimethoprim-sulfamethoxazole, sulfonamides, trimetroprim, chloramphenicol, fosfomycin, and fosfomycin trometramol in reUTI isolates (Figure 1). Classification by resistance type showed that 27% (102/378) of the isolates were included in the XDR group and 59% (223/378) in MDR, and 14% (53/378) were considered still sensitive strains. A Drug-resistance score was used considering the total number of antimicrobials to which each isolated presented resistance (18). In this regard, UPEC strains from reUTI and peUTI did not show significantly different antibiotic resistance (*p* > 0.05) (Figure 2). However, strains isolated from children with peUTI were more resistant than isolates from children with reUTI (*p* < 0.05) and the same was observed for peUTI (*p* < 0.05) and reUTI (*p* < 0.05) in adults.

### 3.3 Phylogenetic diversity

Phylogenetic analysis of the *E. coli* strains showed that 96.4% (380/394) were included in one of the 4 phylogroups evaluated and only in 3.6% (14/394) of the isolates was not possible to define the group. Forty-four percent of the strains corresponded to phylogroup B2, 27% to A, 14.8% to D and 14% to B1; when performing a probability analysis to know the relationship of the phylogroup between patients with peUTI versus reUTI it was found that B2 was the most common in strains from patients with persistence (*p* < 0.0001) and A in isolates from patients with reinfections (Table 2).

**Table 2:** *Escherichia coli* phylogroups prevalence in UPEC from peUTI and reUTI.

Phylogroup	peUTI, *n* = 117	reUTI, *n* = 263	Total *n* = 380	*p*-value
A	0	104 (39.5%)	104 (27%)	–
B1	0	53 (20.2%)	53 (14%)	–
B2	114 (97.4%)	52 (19.8%)	166 (44%)	**<0.0001**
D	3 (2.6%)	54 (20.5%)	57 (14.8%)	–

Fisher’s exact test was performed, only statistically significant *p*-values are shown.

### 3.4 Virulence genes

**Figure 3:**
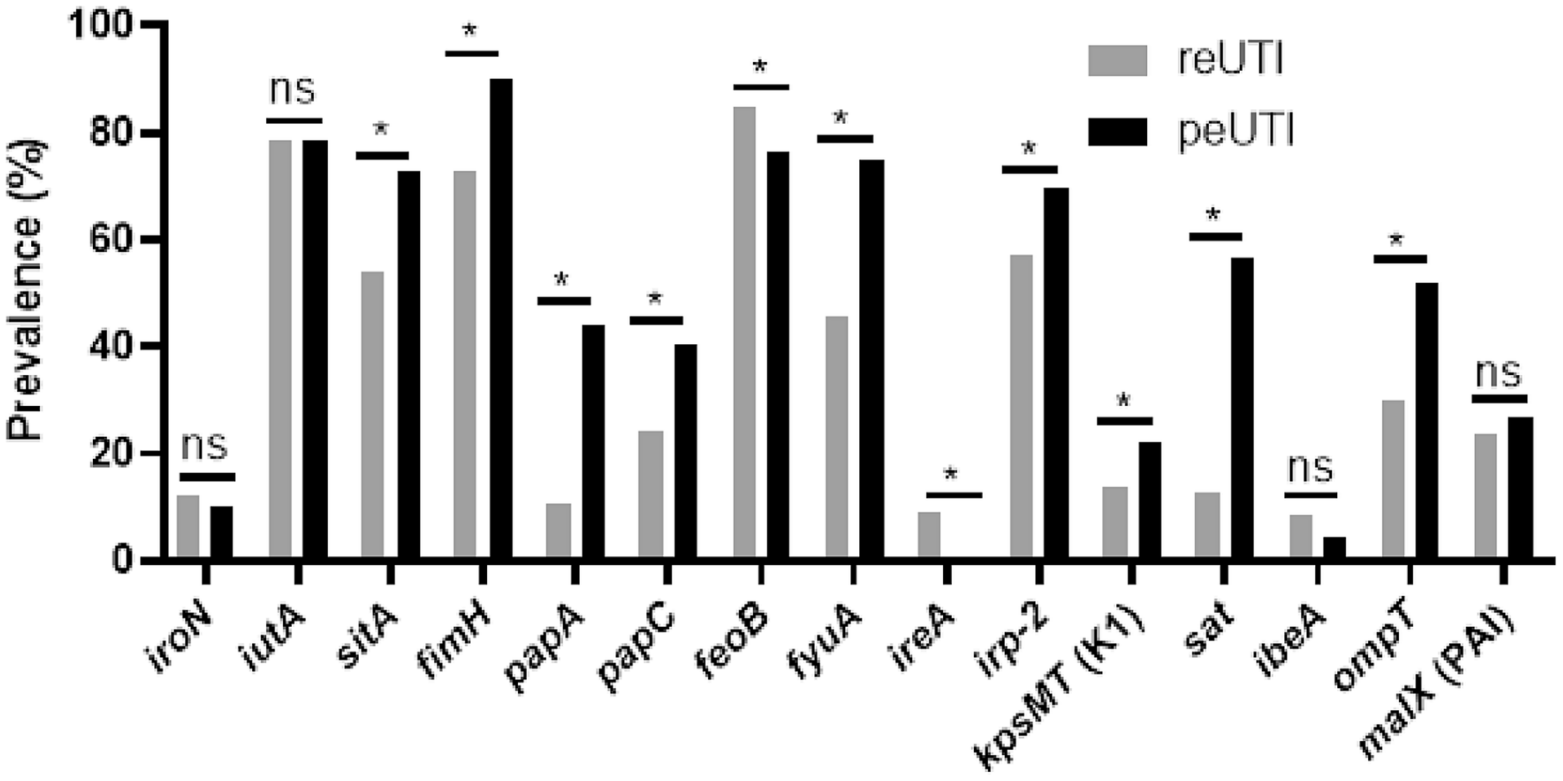
Virulence traits in UPEC isolates from patients with peUTI or reUTI. Fisher’s exact test was performed, ns, statistically non-significant and *p < 0.05.

**Figure 4:**
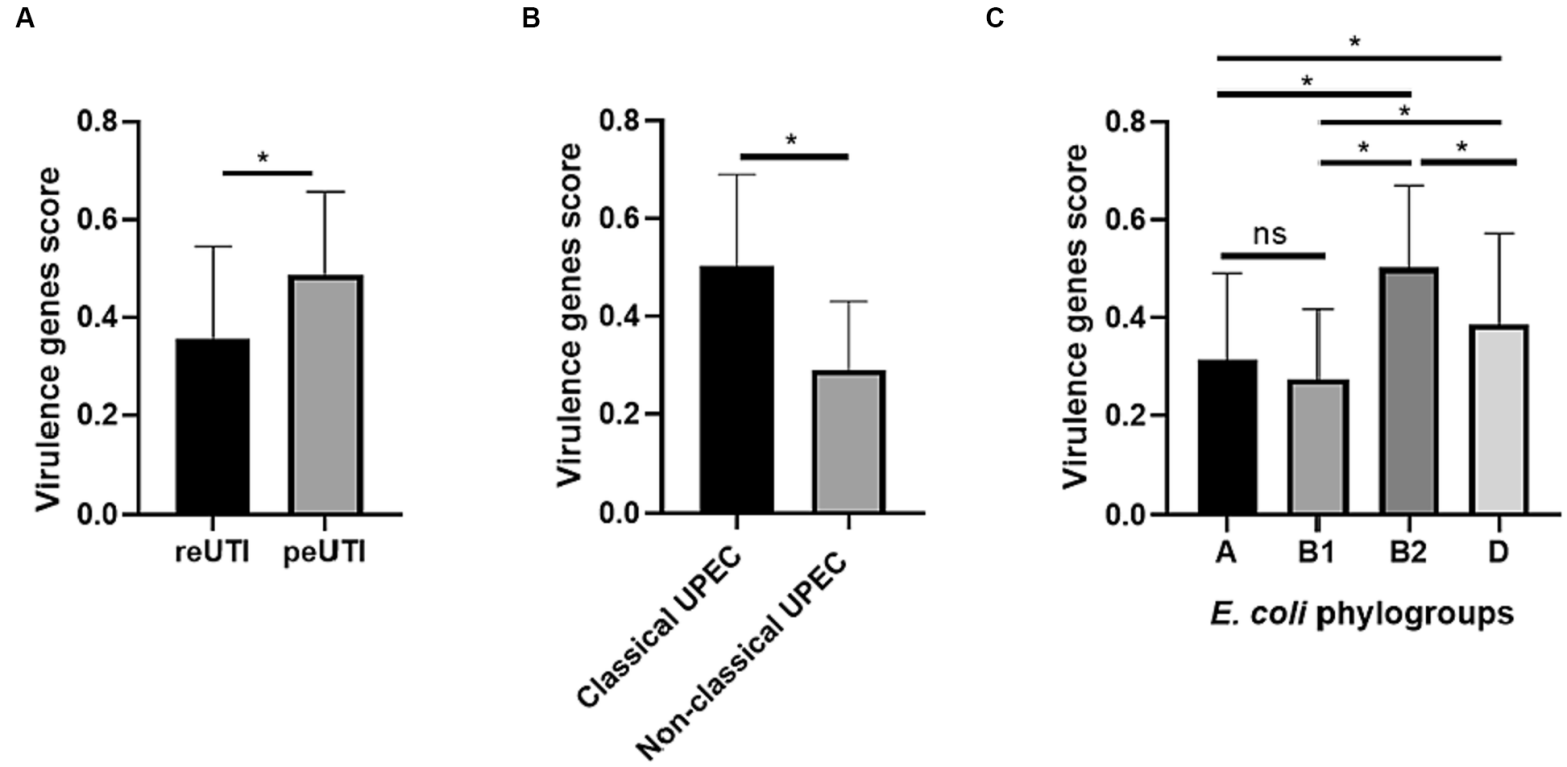
*Escherichia coli* isolates from complicated UTI. **(A)** Virulence genes score in strains from reUTI and peUTI, **(B)** Classical and Non-Classical UPEC groups, and **(C)** *E. coli* phylogroups. Student’s *t*-test was performed, ns, statistically non-significant and *p < 0.05.

The assay to identify virulence genes (Figure 3), showed the presence of 6 genes (*iutA*, *sitA*, *fimH*, *feoB*, *fyuA*, and *irp-2*) in more than 50% of the strains analyzed. *fimH*, *papA*, *papC* (adhesins), *sitA*, *fyuA*, *irp-2* (iron scavengers), *kpsMT*, and *ompT* (protectins) and sat (self-transported cytotoxin) were identified more frequently in strains isolated from peUTI patients (*p* < 0.05). On the other hand, *feoB* and *ireA* were observed more frequently in patients with reUTI (*p* < 0.05). To define whether the presence of the genes favors the persistence of the bacteria carrying them, virulence score analysis was used. This analysis showed that UPEC strains isolated from patients with peUTI presented a higher number of virulence genes (*p* < 0.0001), compared to strains from reUTI (Figure 4A); particularly, classical UPEC strains showed a higher virulence score regardless of the type of cUTI (*p* < 0.0001) (Figure 4B). Additionally, it was observed that strains from group B2 contained a higher number of the virulence genes analyzed, than strains included in other phylogroups (B2 vs. A, *p* < 0.0001; B2 vs. B1, *p* < 0.005; B2 vs. D, *p* < 0.05) (Figure 4C). UPEC strains from peUTI and reUTI showed a significant correlation (Pearson’s coefficient = 0.2171; *R*^2^ = 0.04, *p* < 0.0001) between virulence and antibiotic resistance scores.

### 3.5 *In vitro* assays

To corroborate whether the presence of certain genes was involved in the virulence of the strains and in the variety of cUTI (peUTI or reUTI) presented by the patients, *in vitro* tests were implemented to analyze the adherence, invasiveness, and biofilm formation of some of the bacteria included in the study.

#### 3.5.1 Adherence to cells

Adherence capacity was evaluated in 72 strains from 6 patients with peUTI and 3 with reUTI, who were followed up for >3 months. The assay reported 20 (27.7%) adherence to cells with higher prevalence in isolates from patients with peUTI 70% (14/20). The correlation between adherence and serotype showed that 80% (16/20) had the classic UPEC serotypes O25: H4 (10), O75:NM (4), O6:H1 (2) and 4 the serotype O11:H25 (non-UPEC). The analysis of genes identified in the adherent strains reported the presence of *fimH* (38%), *papA* (36%) and *papC* (31%). As previously mentioned, *fimH* and *papC* were mainly identified in isolates from peUTI patients (*p* < 0.05).

#### 3.5.2 *In vitro* invasiveness

To evaluate cell invasiveness, 54 strains were selected from peUTI and patients with reUTI. The assay showed that 37% of the strains were invasive, with a higher prevalence of 12/20 (60%) in isolates from patients with peUTI. Association analysis between invasiveness and serotype showed that 16 (80%) of the isolates belonged to the classical UPEC serotypes [O25:H4 (10), O6:H1 (4), O4:H5 (2)] and 4 (20%) the non-UPEC serotypes [O28ab:H4 (2), O45:HND (2)]. The quantitative invasiveness assay showed that the number of invasive bacteria isolated from peUTI was higher (2.8×10^3^ CFU/mL) than those isolated from patients with reUTI (*p* < 0.05). *E. coli* O25:H4 strains were the most invasive (1.2×10^3^-7×10^3^ CFU/mL) reporting a logarithm higher than that obtained in *Shigella boydii* 21,639 (5×10^2^ CFU/mL) and *E. coli* 1,124 (3×10^2^ CFU/mL) strains used as controls.

#### 3.5.3 Biofilm formation

Eighty strains isolated from 13 patients with cUTI were selected to evaluate biofilm formation, 29 were from 5 patients with peUTI and 51 from 8 with reUTI. The results reported a minimum absorbance of 0.2722 and a maximum of 0.6694, with a cut-off point of 0.339. With these data it was defined that 29/80 (36%) formed a biofilm classified as weak after 72 h of incubation, 12 strains were isolated from 3 patients with peUTI and 17 from 5 patients with reUTI, with no significant differences (*p* > 0.05). However, when performing the relationship between serotypes of the strains it was found that 17 (59%) belonged to serotypes O25:H4 (8), O4:H5 (5), O6:H1 (4) classic UPEC, and 11 (38%) to serotypes O11:H25 (2), O12:NM (4), O45:H (5) and one non-defined (ND), the statistical analysis between these classic and non-classic groups gives a significant value (*p* < 0.05). Ag43 is a membrane protein associated with biofilm formation identified in 26 *E. coli* strains, thus it was important to identify the flu alleles related to Ag43 expression. Gene analysis showed the presence of *fluA* and *fluB* in both UPEC-CFT073 and the 80 strains selected for biofilm formation, *fluB* was the most prevalent (83%) in strains that formed biofilms (*p* < 0.05) (Table 3).

**Table 3:** Biofilm formation and genes profile in *E. coli* isolates from peUTI and reUTI.

Gen	Biofilm producers, *n* = 29	Non-biofilm producers, *n* = 51	Total *n* = 80
*fluA* (Ag43a)	5 (17%)	3 (6%)	8 (10%)
*fluB* (Ag43b)	22 (76%)	8 (16%)	30 (37%)
*fluA/fluB*	2 (7%)	1 (2%)	3 (4%)
No genes	0	39 (76%)	39 (49%)

## 4 Discussion

*Escherichia coli* strains are the most common bacteria isolated from urine of patients with acute and cUTI infections. In the cases where cUTI is caused by the same microorganism the infection is considered a peUTI, while in cases where the isolated microorganisms are different to those identified in the previous sample the infection is considered a reUTI. To define the characteristics of the *E. coli* that is causing the cUTI the isolate could be characterized by phenotypic (serological and biochemical typing) or genotypic (ribotyping, pulsed-field electrophoresis or MLST) methods (27–31). As previously mentioned, our work group has the complete sera scheme to perform the antigenic characterization of *E. coli*, which allows us to define the type of cUTI, *i. e*. peUTI or reUTI.

In two previous studies patients with cUTI were followed up for 7 to 18 months, where *E. coli* strains were isolated in approximately 70% of the samples (13, 14). Serotyping of *E. coli* isolates from some patients with cUTI, showed O25, O75, O6 serogroups (classic UPEC) in the different urine cultures evaluated, because the serogroup in each patient was the same always, the type of infection was classified as peUTI, same observations have been reported by other authors (29, 32, 33). However, it was interesting to observe in a patient with peUTI the identification of the O9 serogroup, not included in the classic UPEC pathotype and considered by other authors as enterohemorrhagic *E. coli* (34). This circumstance raises the possibility that gene transfer from UPEC to strains such as O9 is taking place in the intestine, favoring their survival in new environments such as the urinary tract (18, 35). When analyzing the data obtained during patient follow-up from it was found that initially the patient only presented reUTI, however, after several isolations of different *E. coli* serogroups O9 strains were identified and being isolated in the following urine cultures the infection changed to a peUTI. Furthermore, the genetic analysis of these O9 strains, showed the presence of genes linked to iron uptake (*sitA*, *feoB*, and *irp2*), which were also present in greater proportion in the classic UPEC strains (O25, O75, and O6). On the other hand, in reUTI cases a greater diversity of serogroups was identified, which in turn were different in each urine culture. Apparently different aspects may be related to cases of reUTI that favor infection by *E. coli* from the intestinal biota via cross-contamination (36). Although these patients can become free of infection when treated with antimicrobials, the predisposition to become infected favors a new strain to colonize them, causing the reinfection of the condition (37).

The phylogenetic distribution among the study strains was higher toward phylogroups B2 and A, this result contrasts with that reported by other authors, who mention that extraintestinal *E. coli* strains belong mainly to phylogroups B2 and D (18, 38). However, studies conducted in Russia, China, Iran, Portugal, Venezuela, and Mexico, reported similar results to those obtained in this study (13, 39–44). Phylogenetic variation in UPEC strains may be associated with host particularities such as the geographical area, climate and diet type in which they inhabit (45). Other predisposing factors such as anatomical alterations, metabolic diseases, immune status, and hygienic habits would allow gut commensal strains to become opportunistic pathogens (46). The results reported here confirmed that phylogroup B2 is particularly associated with peUTIs; in this regard, Thänert et al. (47) reported the presence of this type of strains preferentially associated with peUTIs, suggesting they might be colonizing the urinary tract, the intestine, or both habitats according to their ability for adaptation. Likewise, in a study on the virulence of strains belonging to phylogroup B2, using a murine model, it was reported that urine isolates are more virulent than those from feces (48). However, studies in France and Sweden evaluating the intestinal microbiota of children and adults, identified phylogroup B2 as carrying virulence genes associated with UPEC strains, which would corroborate that B2 phylogroup strains, despite being commensal, would be able to become extraintestinal pathogens (18, 49).

Virulence plays a very important role in the interaction with the host, in UPEC it has been documented that the number of virulence factors is proportional to the pathogenic potential and would facilitate the colonization of the urinary tract (50–52). In this study, it was observed that virulence-related genes of uropathogenic strains were more prevalent (mean virulence score: 0.501994 ± 0.1673) among strains from patients with peUTI than in those associated with reUTI. These genes could be associated with the ability of *E. coli* strains to resist in the urinary tract since it has been reported that the main genes associated with persistence are those related to adherence and iron uptake capacity (29, 53). Given the high prevalence of certain virulence factors in patients with peUTI, such as adhesins *papA*, *papC*, and *fimH*; those associated with iron uptake *sitA*, *fyuA*, *irp-2*, and Sat toxin (*sat*), whose cytotoxic and immunomodulatory effect has been associated with the survival and the potential generation of bloodstream infections and sepsis in producer strains (54), these could be used as a target for diagnosis or vaccine development, as proposed by Mobley and Alteri (55). On the other hand, it was observed that strains with classic UPEC serogroups and B2 and D phylogroups carried the highest number of virulence genes, similar observations documented by other authors (50, 56).

In the present work, employing immortalized HEp-2 larynx cell line, used as the gold standard to observe adherence patterns of diarrheagenic *E. coli* (57, 58), a low number of UPEC isolates showed diffusely adherent phenotype (59–61), although, in other works an adherence pattern of aggregative phenotype was reported (62). The ability of UPEC strains to adhere and invade bladder epithelial cells in the host has been related to the expression of different fimbrial adhesins (63). In this work, it was found adherent strains harboring *fimH*, *papG*, or *papA*, genes related to type I and type P adhesins, while the rest of the identified adherent strains could be associated with other adhesins such as type S, Curli and the aggregate-forming pili identified in hybrid UPEC/DAEC strains (63–66). In a previous study, we documented that *E. coli* strains could persist >6 months in the urinary tract of patients (14), a recent study by Hidad et al. (31) reported that *E. coli* strains were identified in their investigation for more than 1 year. In the present study, it was identified that 60% of the invasive strains were isolated from peUTI patients, associated with classical UPEC serogroups. Andersen et al. (67) reported that these UPEC strains can re-emerge from infected cells and invade adjacent cells.

Different studies indicate that cUTI (peUTI and reUTI) are associated with biofilm formation and antibiotic resistance (30, 68–70). In this study, weak *in vitro* biofilm formation was observed, where only 36% of the strains were positive. However, a relevant aspect to note is the presence of the b allele of Ag43, related to good biofilm formation (71), identified in 83% of the producing strains. Although the result contrasts with that reported by other authors who mentioned a high percentage of biofilm-forming UPEC strains (69, 72), it is likely that the results we obtained are related to the technique used, in which we perform energetic washes that could be detaching the biofilm.

Antibiotic prophylactic treatment is common in patients with cUTI (73), this favors the selection of uropathogenic resistant strains especially to those used as first choice (74). Although the CLSI only recommends sensitivity to five compounds (cefazolin, sulfonamides, trimethoprim, fosfomycin, and nitrofurantoin) for UPEC strains (16), given the conditions in our country due to the indiscriminate use of antibiotics and the fact that these are enterobacteria, it was decided to test for sensitivity to 32 antimicrobials. The results in this regard showed MDR in *E. coli* strains isolated from both reUTI and peUTI, mainly those belonging to the penicillin family, cephalosporins, sulfonamides and trimetroprim, a similar result reported in other countries (31, 75, 76). However, the *E. coli* from peUTI and reUTI demonstrated >80% susceptibility to certain antibiotics (mecilanam, piperacillin-tozobactam, cefoxitin, meropenem, nitrofurantoin, amikacin, chloramphenicol, fosfomycin, and fosfomycin-trometramol), which could be used empirically, with exceptions, according to the Infectious Diseases Society of America (IDSA) criteria (77). Relevantly, this MDR capability has also been described in *E. coli* strains from the intestine, the main reservoir of UPEC, and an explanation of how this resistance is transmitted between *E. coli* strains to potential UPEC (78). It is because of the rapid spread of MDR bacteria, that WHO mentioned *E. coli* as a critical priority pathogen for the development of new treatments (1).

Different authors have mentioned blueberries, D-mannose, probiotics or surgical treatments in patients with urinary tract complications as potential treatment alternatives (74). In two prospective studies conducted previously by our group bacterial lysates (autovaccines, i.e., immunostimulants) were used, and the remission of the clinical picture and control of the infection for periods ranging from 6 to more than 18 months was achieved (13, 14). However, improvement was only observed in patients with reUTI, which again confirms the presence of some other properties in the strains that cause peUTI. Some other alternatives for the treatment and control of cUTI should be developed, in the laboratory we are working with lysates prepared with the strains that were most frequently isolated in the prospective studies carried out. The preliminary results obtained in an animal model are encouraging; however, there is still a long way to go.

According to the results, the strains isolated from peUTI patients are professional bacteria in the generation of cUTI, with genetic and phenotypic characteristics of uropathogenic strains, however, the strains isolated from patients with reUTI correspond to strains of *E. coli* from the intestinal biota that have acquired part of the genetic information that allows them to reach the Urinary Tract and produce UTI but do not remain permanently. This is why they can be eliminated with antimicrobial treatment; however, due to factors related to the host, patients are reinfected but with a different microorganism each time, thus generating a reUTI.

## Data availability statement

The original contributions presented in the study are included in the article/Supplementary material, further inquiries can be directed to the corresponding author/s.

## Author contributions

UH-C and CAE: study conception and design. UH-C, AN-O, MEC-B, JM-L, LMR-R, and AN-C: data collection. UH-C, REA-C, and CAE: draft manuscript preparation. UH-C, REA-C, and CAE: analysis and interpretation of results. All authors reviewed and approved the final version of the manuscript.
