# Supplementary material for: Phenotypic and genotypic characteristics of Escherichia coli strains isolated during a longitudinal follow-up study of chronic urinary tract infections
Source: Front Public Health. 2023 Nov 22;11:1240392. doi: 10.3389/fpubh.2023.1240392 (PMC10702777; doi:10.3389/fpubh.2023.1240392)
Supplement: Supplementary file 1 [file Data_Sheet_1.PDF]

Table S1. Primers sequences for the amplification of virulence genes

Genes	Sequence (5'-3')	Tm	Size of amplicon(bp)	*Reference*
feoB	F aattggcgtgcatgaagataactg<br>R agctggcgacctgatagaacaatg	59 ºC	470	Runyer-Janecky *et al* 2003
sitA	F agggggcacaactgattctcg<br>R taccgggccgttttctgtgc		608	
ireA	F gatgactcagccacgggtaa<br>R ccaggactcacctcacgaat	63 ºC	254	Rodriguez-Siek., 2005
fimH	R agggggacatatagcccccttc<br>F tcgagaacggataagccgtgg		508	
irp-2	F aaggattcgctgttaccggac<br>R tcgtcgggcagcgtttcttct	63 ºC	287	
kpsMTII**	F tagcaaacgttctattggtgc<br>con kpsMT II-Reverse		153	
papC	F gtggcagtatgagtaatgaccgtta<br>R atatcctttctgcagggatgcaata	52 ºC	205	
fyuA	F tgattaaccccgcgacgggaa<br>R cgcagtaggcacgatgttgta		787	
ibeA	F aggcaggtgtgcgccgcgtac<br>R tggtgctccggcaaaccatgc	57 ºC	171	
iutA	F ggctggacatcatgggaactgg<br>R cgtcgggaacgggtagaatcg		302	
papA	F atggcagtggtgttttggtg<br>R cgtcccaccatacgtgctcttc	52 ºC	717	
ompT	F atctagccgaagaaggaggc<br>R cccgggtcatagtgttcatc	51.6 ºC	559	
iroND	F aagtcaaagcaggggttgcccg<br>R gacgccgacattaagacgcag	53 ºC	667	
malX-PAI	F ggacatcctgttacagcgcgca<br>R tcgccaccaatcacagccgaac	57 ºC	925	
sat	F actggcggactcatgctgt<br>R aaccctgtaagaagactgagc	55 ºC	387	Ruiz J., 2002

F. Foward, **R**. Reverse
